# Injuries Caused by Ill-Fitting Saddlery and Harness1Read at the summer meeting of the Lincolnshire Veterinary Medical Society, on June 19, 1902.

**Published:** 1902-11

**Authors:** Joshua A. Nunn

**Affiliations:** Veterinary Lieutenant-Colonel


### INJURIES CAUSED BY ILL-FITTING SADDLERY AND HARNESS.[1]

By Joshua A. Nunn,
VETERINARY LIEUTENANT-COLONEL.

When I was asked to read this paper I was somewhat at a loss to determine on a subject, but finally selected this, namely, "Some Harness and Saddle Injuries." I feel I owe an apology in bring-

[1] Read at the summer meeting of the Lincolnshire Veterinary Medical Society, on June 19, 1902.

ing such a matter before you, as, strictly speaking, it is not one within the scope of veterinary science; but, nevertheless, to my mind, it is an important one, and one that among a certain section of our brethren it is, I am afraid, the fashion to ignore and look down upon.

Gentlemen, we all of us aspire to be successful, and in this world to 99 per cent. of its inhabitants success is spelt by "money," and in our own profession I maintain to be successful we must not only be scientific practitioners but horsemen, and be able to show the horsey owner that we know as much and a little more of horse matters than he and his confidential studgroom put together, and that we can occasionally teach the latter worthy a trick or two at his own calling. It is my experience that such little things give the public confidence in the professional man, and although such matters may not be directly profitable they are the bait that brings the big fish to the net, and I therefore consider an acquaintance with the calling of the saddler of as much importance to us as is that of the blacksmith.

Into the construction of saddles and harness I will not enter, as you are acquainted with these matters, but will at once go on to describe the various injuries caused by ill-fitting and faulty parts. Before entering into detail I will remark that certain faults in the gear produced certain injuries, and that a person conversant with such can almost at a glance tell where the fault lies.

In the proper fitting of a saddle the true secret of success is that the whole of the weight-bearing portion should accurately correspond to the surface of the back, or, in other words, that the whole of the weight-bearing surface should be utilized. As you are well aware, a saddle cannot be put far forward on the withers, or it will pin in and interfere with the motion of the shoulder-blades, causing the horse not only to go short and pottering and to stumble, but also very likely cause abrasions on the sides and top of the withers from the points of the tree and gullet-plate. Also the saddle cannot be put far back on the loins, as the part is not adapted to carry weight, and the large propelling muscles would be interfered with. It, therefore, follows that there is only a limited space in the length of the back available, a weight-bearing surface of about twenty-two inches, and this is further reduced if we remember that it is only the flat surface of the rib between the backbone and curve that can be made available. If this is remembered, it can be realized how important it is that every available portion of the bar of the saddle should carry its due proportion of weight.

A position in which an abrasion is seen is on the centre of the spine just under the cantle, and, although it may be caused by the cantle-plate, in the majority of cases I think it is due to too short a saddle, the weight of the rider coming right back on the cantle. It is aggravated by the motion of the horse wrinkling up the skin of the loin at every step he takes, and this being forced against the saddle, which is pressed downward by the rider's position, gives rise to an abrasion. The obvious remedy is to use a saddle-weight, the tree.

Both longitudinally and transversely across the bars on the under surface pieces of webbing are fixed—the webs of the saddle. There is considerable art in adjusting these, and it is a long process, as if not properly done they will stretch and allow the weight of the rider to come on the centre of the backbone, where the tops of the vertebral spines are only covered with skin, and the certain result is a sore.

At the side of the swell of the tree a pad, the bellies, are fixed, over which the upper thighs of the rider come. If not properly stuffed a wound will be caused over the swell of the last two or three ribs. Although not important to the horse, the proper fitting of the bellies is one of some importance to the comfort of the rider, for if not adjusted to his seat and length of thigh it will cause him to sit in an uncomfortable position, either too far forward or back. This will not only give rise to an annoying chafe on the inside of the thigh, but if he rides over a fence is extremely likely to cause sprain of the rider's muscle, a serious injury that has put an end to many a good man's hunting days.

Under the pommel or head of the saddle is an arched piece of steel, the gullet-plate. Owing to the wedge shape of the withers and the weight of the rider pressing the V-shape of the saddle down on it, there is almost more strain on the gullet than elsewhere, and it is highly important that the plate should be of good steel. In some military saddles some years ago this was made of iron, and the result was that under the strain the tree spread and came down on the top of the withers, causing a gall. It is a matter of some controversy whether the head should be straight or cut back; both have been in fashion and both have their advocates. The straight head is the strongest, and is further away from the rider's fork if by accident he is pitched forward. The cut-back is perhaps not so likely to injure the withers if he "comes down," and it is a favorite with some dealers, as it shows off the slope of the shoulder better.

It is important that the points of the tree should not be too narrow, or they will press on the sides of the withers just below the upper and posterior angle of the scapula, and a gall will be caused in that situation. In fact, all the weight should be taken on the part below the junction of the bars, none above it. If the sides of the shoulders are pressed upon or pinned in, not only will there be a gall, but free motion is interfered with, and the horse is stilted in his action and likely to stumble. I have proved this many times over in the case of troop horses. A military saddle has the bars prolonged in front of the fore arch or gullet into "burrs" designed to carry the wallets, and which of necessity press on the top of the shoulder-blade, and I have frequently found that horses that trip and stumble in a military saddle go safely in an ordinary hunting one. I give you the hint, gentlemen, that when consulted as to a horse stumbling it is advisable to look at the saddle and gear as well as the shoes, as this is a point often forgotten.

The bars are, or should be, made of seasoned beechwood, but from a variety of causes they may be bent or distorted, what the saddler terms "cast," in either downward or upward direction. If cast up—that is to say, the ends being turned upward—the lower surface of the bar will be too curving, and all the weight will be taken on the centre; a sore will be the result over the swell of the middle ribs. If in the other direction, all the weight will be on the ends, and a sore made at the side of the withers at the point of union between the bar and gullet and over the swell of the last three or four ribs.

A troublesome sore is often seen on the off side of the withers from a side saddle. From the position a woman naturally leans, to the the near side, and, being seated higher above the back than a man, there is naturally more play; furthermore, all this is aggravated if the lady is not a good horsewoman and cannot sit square; the saddle is naturally drawn over to the near side, and the point of junction of the bar and gullet on the off presses against the side of the withers. I have found that a broad racing surcingle passing right over the seat of the saddle will greatly help to steady it, even with an indifferent horsewoman; but as it is not the fashion to use them, I suppose it is useless to suggest them to our lady clients, especially if they discovered that in it there was a reflection on their horsewomanship.

These, gentlemen, are the chief injuries from the saddle itself, and I will finish this portion of the paper before I go on to speak of others due to certain portions of gear.

All that I have mentioned are caused by the tree not being properly fitted, or, in other words, by it not being adapted to the lines of the back. Now, no doubt to a certain extent, these defects can be remedied by properly adjusted stuffing; still this is objectionable. The stuffing and panels should be looked on only as a cushion to prevent injury from the hard tree, and should be no thicker than is necessary for this purpose; if they are, not only is the weight increased, but the rider, being high above the back, does not get a proper grip and firm seat, but the saddle will roll about to a greater degree, even with a good horseman.

In these injuries a common device to prevent recurrence, or to work the horse, is to "chamber" the saddle. To be effective, the stuffing must be fairly thick. If it is not the centre of the chamber will touch the sore. The hollow must also be of considerable size, which is a point which most saddlers overlook; and above all things it must be made exactly over the injured spot. The usual practice is to put the saddle on the horse without girthing it up, then raise it up and make a mark where it is presumed it will touch the sore, and hollow it out. The result is not usually satisfactory, and it is easily proved by any one who takes the trouble that it is extremely difficult by the eye alone to mark the right spot off.

When it is desired to chamber a saddle the abraded part should be surrounded with a ring of some colored substance, such as soot and vaseline, and the saddle put on and girthed up by the person who is in the habit of doing it. The rider who has given the sore back should then mount the horse and move him off a few steps; when the saddle is taken off the offending spot will be marked clearly, and will be found, as a rule, not to be in the place that would have been fixed upon by the eye only. After the chamber has been made the same process should be gone through, and if the hollow is not soiled the work can be looked upon as satisfactory.

In fitting a saddle for any alteration it is important that the person who is in the habit of doing so should put it on, for in many cases faulty or careless saddling is all that is at fault; also, that the person who is in the habit of riding the horse should mount him, as there is a great difference in men's seats.

If the panel is thin and the rider grips close, the buckle of the girth may give rise to a gall. The best plan in this case I have found is to use a long girth strap and a short girth, so that the buckle comes down below the edge of the flap away from the pressure of the leg. The girth, if a web one, can be shortened by stitching a fold or tuck in it, but there ought to be a leather tongue under the buckle to protect the skin.

Some horses, particularly slab-sided ones with pinned-in elbows, will girth-gall in spite of every contrivance. In England web girths are generally used, and in this case the substitution of a cape raw-hide or a split-leather girth will often overcome the difficulty, especially if one or more of the strands be tied back so as to be clear of the sore place. In cases of necessity it is possible to work the horse with one girth fastened to the D to which the breast-plate goes, the other to those on the belly of the saddle. Such a contrivance is, of course, not very secure, and is somewhat unsightly. The old contrivance of a piece of sheepskin sewn to the girth is effective, but is often put on and neglected, the wool getting rubbed into a hard mass. It should be carefully dried after use and frequently combed and brushed. I have found a soft piece of numdah answer as well, and is not so unsightly.

If the breast-plate is too tight galls will take place between the forelegs, particularly if the horse is galloped. The obvious remedy is either to leave the breast-plate off or to loosen it. It is, of course, only caused by great carelessness; but, nevertheless, I have been before now consulted about such things. In such a case it is absolutely necessary that the man who usually saddles the horse should do so, otherwise it would be almost impossible to find out what was wrong.

I will now go on to speak of harness galls, which, to the majority of us, are more interesting than the saddle. It is an important point to remember that a saddle gall is caused by pressure, a collar gall by friction. In progressing, one foot and the corresponding shoulder, at whatever pace the horse is going, are in advance of the other, and it is only when standing still and "square" that the two shoulders are symmetrical; the result is that the collar is always oscillating from one side to the other. This can be proved by putting one on a horse and walking him past you, when the movement will be easily observed. The rules for fitting a collar are the same as for the saddle, viz., that the whole of the bearing surface should be utilized, and that it should coincide with the curves of the neck. When the way the collar moves is taken into consideration, the reason for this accurate adjustment is obvious, as, if only one point is bearing on the surface, not only will all the pressure be concentrated on it, but the collar, resting as it were on a pivot, will oscillate to a dangerous degree. This gives rise to undue friction, and a gall is the result. Collars can be too long and too short, too wide and too narrow; the terms speak for themselves, and the whole secret of fitting one is to hit off a happy medium, so that it fits close enough to avoid oscillation and friction, but not so tight as to pinch. In adjusting the length, remember that as the horse throws his weight into it the collar rises, so sufficient space must be left that the bottom will not impinge on the windpipe. When on, the collar should fit nicely and evenly; if it has to be forced down it is certain that in some place it is too narrow. If it see-saws a great deal it will have too much stuffing in the centre and not enough above or below. To test this, hold it steady at the top and move it from the bottom, where there should not be play of more than two inches from side to side over the shoulder-joints; if there is, it is too wide. At the bottom there should be space enough to admit of the fingers being inserted between it and the skin. I mentioned that the whole bearing surface should be in apposition to the skin, also that injuries were the result of friction. To this latter rule there is one exception, viz., if the top of the collar rests on the neck a troublesome pressure sore will be caused. This was noticed some years ago among the London Fire Brigade horses that are kept ready harnessed, the weight of the collar causing injuries of this nature. In old collars that get flat the edge of the after whale is likely to make a sore.

The top of the withers, at the spot where I mentioned a sore would take place from the collar hanging on it, is also liable to injury if the horse is used in double harness, by the pole chains being too tight and dragging the collar on to it. This can be avoided by stuffing the collar well below, so that the top will not come in contact with the withers; metal shields are also used for this purpose, and answer well, but the objection to them is that they are liable to be bent and broken, and if put on in this condition will injure the neck more than the collar. The injury on the upper part of the shoulder-blade, slightly to the front, comes from too tight a collar, and either some of the stuffing should be taken out or the collar stretched. It should, however be remembered that if it is stretched it is shortened in corresponding degree. Too loose a collar will cause injury, and, as a rule, it is found where the trace joins the collar. The true shape of the collar that causes this will not be seen till the traces are tightened, as when slack the collar rides forward. If a complaint is made about a collar I think it is almost more important to see it on the horse than a saddle, and he should be put into a trap and made pull. It is no use looking at the collar hanging around his neck to find out what is wrong, as I have seen done before now on many occasions.

These are some of the more important injuries caused by saddlery and harness, but the list is nothing like complete; and the matter of bits and bridles I have not touched upon, as it is a subject in itself.—*Veterinary Journal*, August, 1902.

